# Identification of α-galactosylceramide as an endogenous mammalian antigen for iNKT cells

**DOI:** 10.1084/jem.20240728

**Published:** 2024-12-20

**Authors:** Yuki Hosono, Noriyuki Tomiyasu, Hayato Kasai, Eri Ishikawa, Masatomo Takahashi, Akihiro Imamura, Hideharu Ishida, Federica Compostella, Hiroshi Kida, Atsushi Kumanogoh, Takeshi Bamba, Yoshihiro Izumi, Sho Yamasaki

**Affiliations:** 1 Department of Molecular Immunology, Research Institute for Microbial Diseases, Osaka University, Suita, Japan; 2 Laboratory of Molecular Immunology, Immunology Frontier Research Center, Osaka University, Suita, Japan; 3 Department of Respiratory Medicine and Clinical Immunology, Graduate School of Medicine, Osaka University, Suita, Japan; 4 Department of Systems Life Sciences, Graduate School of Systems Life Sciences, Kyushu University, Fukuoka, Japan; 5 Department of Applied Bioorganic Chemistry, Gifu University, Gifu, Japan; 6 Institute for Glyco-core Research, Gifu University, Gifu, Japan; 7 Department of Medical Biotechnology and Translational Medicine, University of Milan, Milano, Italy; 8 Department of Respiratory Medicine, National Hospital Organization Osaka Toneyama Medical Center, Toyonaka, Japan; 9 Department of Immunopathology, World Premier International Research Center Initiative, Immunology Frontier Research Center, Osaka University, Suita, Japan; 10 Integrated Frontier Research for Medical Science Division, Institute for Open and Transdisciplinary Research Initiatives, Osaka University, Suita, Japan; 11 Center for Infectious Disease Education and Research, Osaka University, Suita, Japan; 12 Center for Advanced Modalities and DDS, Osaka University, Suita, Japan; 13 Division of Metabolomics, Medical Research Center for High Depth Omics, Medical Institute of Bioregulation, Kyushu University, Fukuoka, Japan

## Abstract

Invariant natural killer T (iNKT) cells are unconventional T cells recognizing lipid antigens in a CD1d-restricted manner. Among these lipid antigens, α-galactosylceramide (α-GalCer), which was originally identified in marine sponges, is the most potent antigen. Although the presence of α-anomeric hexosylceramide and microbiota-derived branched α-GalCer is reported, antigenic α-GalCer has not been identified in mammals. Here, we developed a high-resolution separation and detection system, supercritical fluid chromatography tandem mass spectrometry (SFC/MS/MS), that can discriminate hexosylceramide diastereomers (α-GalCer, α-GlcCer, β-GalCer, or β-GlcCer). The B16 melanoma tumor cell line does not activate iNKT cells; however, ectopic expression of CD1d was sufficient to activate iNKT cells without adding antigens. B16 melanoma was unlikely to generate iNKT cell antigens; instead, antigen activity was detected in cell culture serum. Activity-based purification and SFC/MS/MS identified dihydrosphingosine-based saturated α-GalCer as an antigenic component in serum, bile, and lymphoid tissues. These results show the first evidence for the presence of potent antigenic α-GalCer in mammals.

## Teaser

α-Galactosylceramide (α-GalCer), a marine sponge-derived canonical antigen for invariant natural killer T (iNKT) cells, has not been detected in mammals. In this study, we report the molecular detection of α-GalCer in mammals using supercritical fluid chromatography tandem mass spectrometry.

## Introduction

Invariant natural killer T (iNKT) cells are a unique subset of unconventional T cells that are highly conserved in mammals. iNKT cells express invariant TCRs (mouse, Trav11-Traj18–Trbv13/29/1; human, TRAV10-TRAJ18–TRBV25) that recognize lipid antigens presented by monomorphic CD1d molecules (Brennan et al., 2013). Upon stimulation with lipid antigens, iNKT cells rapidly trigger effector functions to induce various immune responses against infection, internal stresses, or cancers (Slauenwhite and Johnston, 2015). The most potent antigen for iNKT cells is α-galactosylceramide (α-GalCer), which was initially extracted from marine sponges (Kawano et al., 1997). The fact that iNKT cells undergo thymic selection and respond to non-infectious insults implies that endogenous ligands may also control iNKT cell function. Several endogenous lipid ligands have been reported (Gumperz et al., 2000; Zhou et al., 2004; Fox et al., 2009; Facciotti et al., 2012), whereas α-GalCer has never been detected in mammalian tissues. Indeed, endogenous GalCer is thought to be present as the β anomer that lacks iNKT ligand activity (Merrill, 2011; Reza et al., 2021; Brennan et al., 2017). Recent papers have reported the presence of a low amount of α-type hexosylceramides (HexCer) in mammals (Kain et al., 2014; Brennan et al., 2014, 2017). However, the discrimination of the endogenous α-anomeric GalCer and glucosylceramide  (GlcCer) has not been fully achieved.

Liquid chromatography mass spectrometry (LC/MS) is a standard analytical technique for determining the trace levels of metabolites or lipids. However, difficulties arise with isomers, which cannot be easily distinguished without additional methodological breakthroughs (Brennan et al., 2017). Indeed, the successful separation of HexCer diastereomers, such as α-GalCer, β-GalCer, α-GlcCer, and β-GlcCer, has not been generalized.

Supercritical fluid chromatography (SFC) is a chromatographic technique that uses a supercritical fluid such as supercritical CO_2_ as a mobile phase (Taylor, 2010). The polarity of supercritical CO_2_ is close to that of *n*-hexane and the polarity of the mobile phase can be significantly changed by adding a polar organic solvent such as methanol. SFC with supercritical CO_2_ has the potential to accurately separate more lipophilic stereoisomers and structural isomers using chiral stationary phase and normal-phase achiral stationary phase (Taylor, 2010; Takeda et al., 2018; Berger, 2022). We therefore applied SFC/tandem mass spectrometry (SFC/MS/MS) to attempt to discriminate HexCer diastereomers in the host.

In the present study, we report the presence of α-GalCer in mammalian tissues by developing a novel analytical platform.

## Results and discussion

### iNKT cell antigen(s) are presented by CD1d on tumor cells

**Figure 1.**
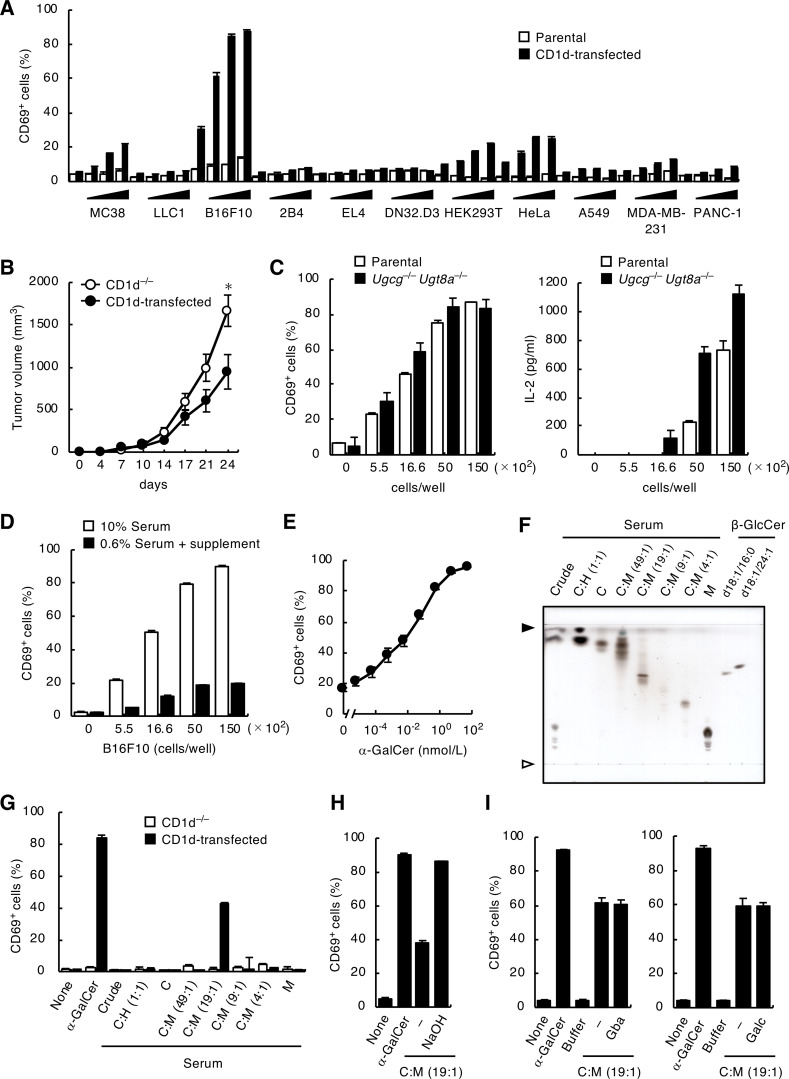
**Serum contains antigens for iNKT cells. (A)** DN32.D3 cells were co-cultured with 5.5 × 10^2^, 1.66 × 10^3^, 5.0 × 10^3^ and 1.5 × 10^4^ parental or CD1d-transduced MC38, LLC1, B16F10, 2B4, EL4, DN32.D3, HEK293T, HeLa, A549, MDA-MB-231, and PANC-1 cells for 16 h and analyzed for CD69 expression. **(B)** 5 × 10^5^ CD1d^−/−^ or CD1d-transduced B16F10 cells were injected subcutaneously into the right flank of C57BL/6J mice (*n* = 8). Tumor volume was measured every 3–4 days. **(C)** DN32.D3 cells were co-cultured with the indicated cell number of WT or Ugcg^−/−^ Ugt8a^−/−^ CD1d-transduced B16F10 cells for 16 h and analyzed as in A (left). Concentrations of IL-2 in the supernatants were measured (right). **(D)** CD1d-transduced B16F10 cells were cultured in RPMI 1640 supplemented with 10% FCS or in RPMI 1640 with 0.6% FCS and 9.4% animal component-free cell culture supplement for 7 days. DN32.D3 cells were then co-cultured with those B16F10 cells for 16 h and analyzed as in A. **(E)** DN32.D3 cells were co-cultured with CD1d-transduced B16F10 cells that were cultured in RPMI 1640 supplemented with 0.6% FCS and 9.4% animal component-free cell culture supplement for 7 days as in D in the absence or presence of α-GalCer (t18:0/26:0) (KRN7000) for 16 h and analyzed as in A. **(F)** Lipids extracted from serum were separated into seven fractions by open column chromatography and analyzed by HPTLC using C:M:W (65:25:4; vol/vol/vol) followed by staining with copper acetate reagent. Commercial β-GlcCer was used as a reference (right lanes). Open and closed arrowheads denote the origin and solvent front, respectively. **(G)** CD1d^−/−^ or CD1d-transduced DN32.D3 cells were stimulated with each fraction separated from serum lipids in F for 16 h and analyzed as in A. α-GalCer (t18:0/26:0) was used as a positive control. **(H)** CD1d-transduced DN32.D3 cells were stimulated with the C:M = 19:1 fraction of serum lipids with or without hydrolysis treatment for 16 h and analyzed as in A. α-GalCer (t18:0/26:0) was used as a positive control. **(I)** CD1d-transduced DN32.D3 cells were stimulated with the C:M = 19:1 fraction of serum lipids treated with Gba (left) or Galc (right) for 16 h and analyzed as in A. α-GalCer (t18:0/26:0) was used as a positive control. Data are presented as mean ± SD (A–E and G–I) and are representative of three independent experiments (A–I). Statistical significance was determined by Student’s *t* test. *, P < 0.05. Source data are available for this figure: SourceData F1.

**Figure S1.**
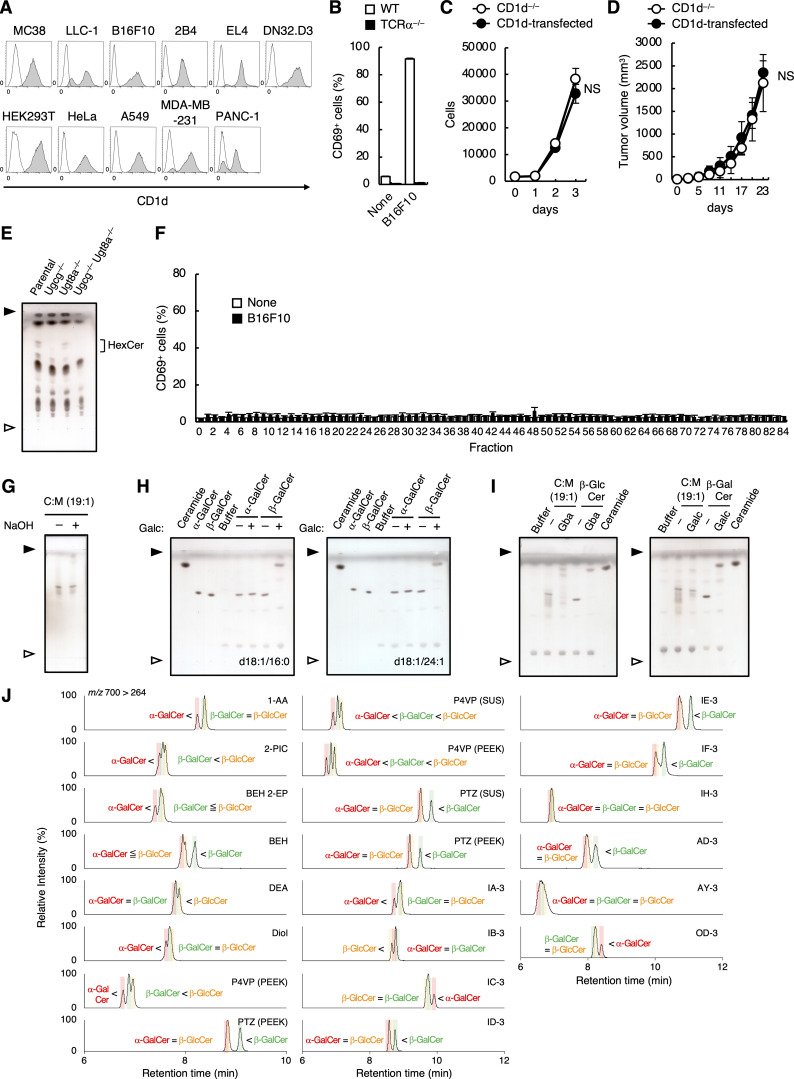
**Serum contains antigens for iNKT cells. (A)** Surface expression of CD1d on CD1d-transduced cell lines. Filled histogram, anti-mouse CD1d antibody; open histogram, isotype control antibody. **(B)** WT or TCRα^−/−^ DN32.D3 cells were co-cultured with CD1d-transduced B16F10 cells for 16 h and analyzed for CD69 expression. **(C)** CD1d^−/−^ or CD1d-transduced B16F10 cells were seeded onto 24-well plates. Growth curves were generated using cell counting by flow cytometer every 24 h. **(D)** 5 × 10^5^ CD1d^−/−^ or CD1d-transduced B16F10 cells were injected subcutaneously into the right flank of Jα18-deficient mice (*n* = 7). Tumor volume was measured every 3–4 days. **(E)** The crude lipids extracted from WT, Ugcg^−/−^, Ugt8a^−/−^, and Ugcg^−/−^ Ugt8a^−/−^ B16F10 cells were analyzed by HPTLC using C:M:W (65:25:4; vol/vol/vol) and stained with copper acetate reagent. **(F)** Lipid extracts from B16F10 cells (5 × 10^6^) were separated into 84 fractions in a 96-well plate by LC-FRC system and evaporated. DN32.D3 cells were stimulated in the 96-well plate for 16 h and analyzed for CD69 expression. Fractionation was performed in triplicate. **(G)** The C:M = 19:1 fraction of serum lipids before and after hydrolysis treatment was analyzed by HPTLC as in E. **(H)** Commercial α- and β-GalCer (d18:1/16:0) (left) and α- and β-GalCer (d18:1/24:1) (right) were treated with Galc and analyzed by HPTLC as in E. **(I)** The C:M = 19:1 fraction of serum lipids and commercial β-GlcCer or β-GalCer were treated with Gba (left) or Galc (right) and analyzed by HPTLC as in E. **(J)** Screening of columns to separate three diastereomers of synthesized HexCer (d18:1/16:0). MRM chromatograms of SFC/MRM analysis using the columns in Table S1 are shown. The MRM transition was set to 700.57 > 264.27 (precursor ions selected as [M+H]^+^). The SFC analysis conditions for 1-AA, 2-PC, BEH 2-EP, BEH, DEA, Diol, P4VP (PEEK), and PTZ (PEEK) (left) were as follows: column temperature, 50°C; mobile phase A, supercritical carbon dioxide; mobile phase B, M:W (95:5, vol/vol) with 0.1% (wt/vol) ammonium acetate; flow rate of mobile phase, 1.0 ml min^−1^; flow rate of make-up pump, 0.1 ml min^−1^; back-pressure regulator, 10 MPa. The gradient conditions were as follows: 1% B, 0–1 min; 1–75% B, 1–24 min; 75% B, 24–26 min; and 1% B, 26–30 min. The SFC analytical conditions for other columns (center and right) were as described above with modification as follows: column temperature, 40°C; gradient conditions, 1% B, 0–1 min; 1–50% B, 1–17 min; 50% B, 17–26 min; and 1% B, 26–30 min. The MRM operating conditions were identical to those of the SFC/MRM analysis method. The colored shadows indicate the peaks coincident with the RT of synthesized α-GalCer (red), α-GlcCer (blue), β-GlcCer (green), and β-GalCer (yellow), respectively. Open and close arrowheads denote the origin and solvent front, respectively (E and G–I). Data are presented as mean ± SD (B–D and F) and are representative of three independent experiments (B–E and G–J). Statistical significance was determined by Student’s *t* test (C and D). NS, not significant. Source data are available for this figure: SourceData FS1.

To establish antigen-presenting cell lines for iNKT cells, we expressed CD1d in several cell lines. Among them, the B16 melanoma expressing CD1d potently activated a hybridoma expressing iNKT TCRαβ without any additional antigens (Fig. 1 A; and Fig. S1, A and B), suggesting that certain ligand(s) were presented on CD1d in B16 melanoma cells. To evaluate the in vivo relevance of these findings, we injected CD1d-expressing B16 melanomas into mice and evaluated tumor growth. At 24 days after injection, tumor size was significantly reduced in mice injected with CD1d-expressing B16 cells compared with B16 cells lacking CD1d, despite comparable in vitro growth of two cell lines (Fig. 1 B and Fig. S1 C). Although tumor growth was increased when Jα18^−/−^ mice were used as recipients, this difference was not observed (Fig. S1 D), suggesting that B16 cells present ligand(s) on CD1d that can activate iNKT cells in vivo.

### Serum contains glycolipid antigens for iNKT cells

To examine the possible contribution of known biosynthetic enzymes for β-GalCer or β-GlcCer to the generation of antigenic α-anomers (Kain et al., 2014), we depleted UDP-glucose ceramide glucosyltransferase (Ugcg) and UDP-galactosyltransferase 8A (Ugt8A) in B16 cells using the CRISPR-Cas9 system. These two enzymes were dispensable for the ability of CD1d-expressing B16 cells to activate iNKT cells (Fig. 1 C), although β-GlcCer and β-GalCer were depleted (Fig. S1 E). We next extracted lipids from B16 cells using chloroform (C):methanol (M) and fractionated into 84 fractions using an LC-fraction collector (LC-FRC) system; however, none of these fractions had detectable antigen activity (Fig. S1 F). These results raise the possibility that ligand components might be provided exogenously, rather than being cell-intrinsic. We thus examined the components of culture media used for iNKT cell stimulation assays. Co-culture of CD1d-B16 cells with iNKT cells using synthetic supplements instead of fetal calf serum severely impaired CD1d-B16 cell-induced iNKT cell activation (Fig. 1 D), although exogenously added α-GalCer normally activated iNKT cells even in this media (Fig. 1 E). We therefore attempted to purify the antigenic components from serum (Fig. 1 F). One fraction C:M (19:1, vol/vol) exhibited specific activity to stimulate iNKT cells in a CD1d-dependent manner (Fig. 1 G).

The activity of this fraction was resistant to the treatment of hydrolysis (Fig. 1 H and Fig. S1 G) and β-specific degrading enzymes, glucosylceramidase (Gba) and galactosylceramidase (Galc) (Fig. 1 I; and Fig. S1, H and I), suggesting that the active component is likely to be an amphiphilic lipid lacking ester bonds and a β-linked glucose/galactose. Thus, the most likely candidate in serum is an α-GlcCer or α-GalCer.

### Detection of α-GalCer (d18:0/16:0) in serum

**Figure 2.**
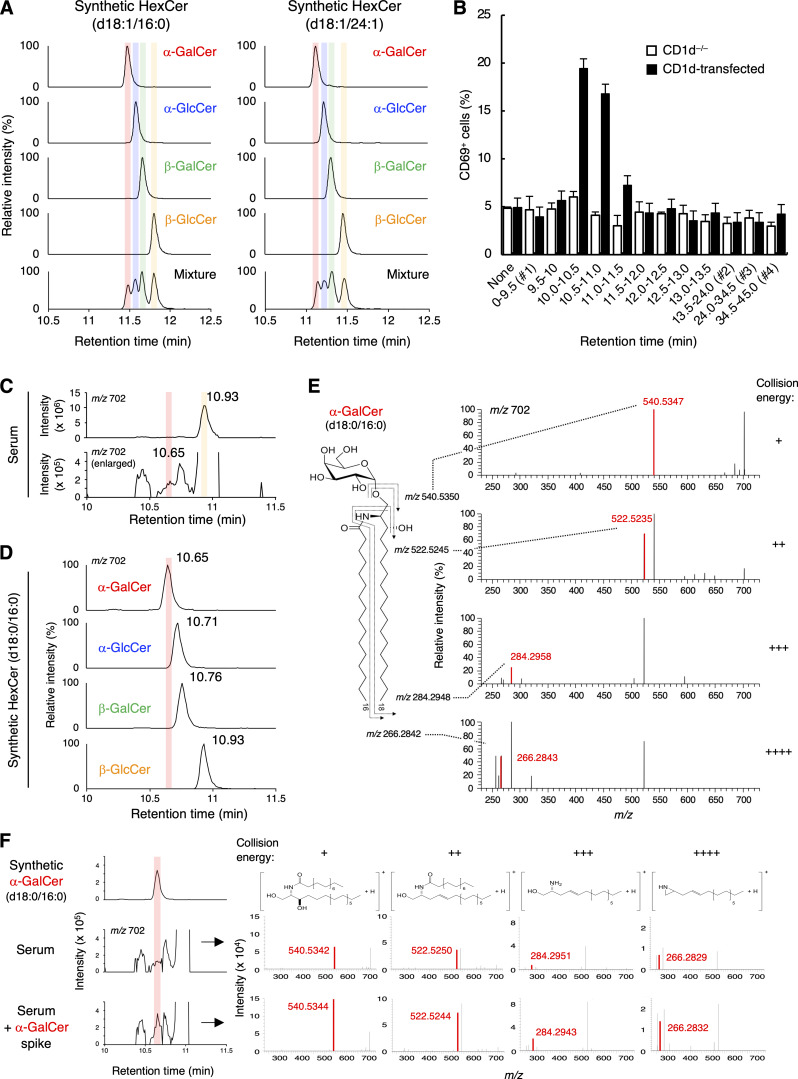
**Separation of HexCer diastereomers. (A)** MRM chromatograms of the synthesized four diastereomers of HexCer (d18:1/16:0) and (d18:1/24:1) were obtained using SFC/MRM. SFC separated the four diastereomers according to their RTs even when mixed (bottom). MRM transitions were 700.57 > 264.27 for HexCer (d18:1/16:0) and 810.68 > 264.27 for HexCer (d18:1/24:1), (precursor ions were selected as [M+H]^+^). The colored shadows indicate the peaks coincident with the RT of synthesized α-GalCer (red), α-GlcCer (blue), β-GalCer (green), and β-GlcCer (yellow), respectively. **(B)** CD1d^−/−^ or CD1d-expressing DN32.D3 cells were stimulated with the C:M = 19:1 SFC-FRC separated fractions from serum lipids for 16 h and analyzed for CD69 expression. The C:M = 19:1 fraction (10 μg) of serum lipids was separated using SFC, and 1/25th of the product was used for 9.5–13.5 min subfractions and 1/75th of the product was used for the other four sub-fractions (0–9.5 [#1], 13.5–24 [#2], 24–34.5 [#3] and 34.5–45 min [#4]) (Fig. S2 A). **(C)** HRMS chromatogram of HexCer (d18:0/16:0) in serum. The colored shadows indicate the peaks coincident with the estimated RT of α-GalCer (d18:0/16:0) (red) and β-GlcCer (d18:0/16:0) (yellow), respectively. **(D)** HRMS-EIC chromatograms of the synthesized four diastereomers of HexCer (d18:0/16:0) obtained using SFC/HRMS. SFC separated the four diastereomers according to their RTs. The red shadow indicates the peak coincident with the RT of synthesized α-GalCer (d18:0/16:0). **(E)** HRMS/MS spectra of candidates for α-GalCer (d18:0/16:0) in serum. From top to bottom, collision energy settings are −10 eV (+), −20 eV (++), −30 eV (+++), and −40 eV (++++). The HRMS/MS of precursor-product ion pair: m/z 702.5878 to 540.5350, 522.5245, 284.2948, and 266.2842, respectively. **(F)** HRMS chromatograms of HexCer (d18:0/16:0) and the HRMS/MS spectra of candidates for α-GalCer (d18:0/16:0) in serum spiked with synthesized α-GalCer (d18:0/16:0). From left to right, collision energy settings are −10 eV (+), −20 eV (++), −30 eV (+++), and −40 eV (++++). The structures of product ions are shown. The red shadow indicates the peak coincident with the RT of synthesized α-GalCer (d18:0/16:0). The mass error tolerance of the precursor ion (m/z 702.5878 as [M+H]^+^) was <7 ppm (E and F). The HRMS/MS of precursor-product ion pair: m/z 702.5878 to 540.5350, 522.5245, 284.2948, and 266.2842, respectively (E and F). Data are presented as mean ± SD (B) and are representative of three independent experiments (A, C, and D–F).

We, therefore, aimed to establish a chromatography method that separates α-GlcCer, α-GalCer, β-GlcCer, and β-GalCer. To this end, we used four diastereomers for major d18:1/16:0 and d18:1/24:1 species, respectively. SFC/multiple reaction monitoring (SFC/MRM) was used to discriminate these four synthetic HexCer diastereomers that have the same side chains (d18:1/16:0) and (d18:1/24:1). After screening of 20 different columns (Fig. S1 J and Table S1), we finally found that the tandem connection of a silica-based poly-4-vinylpyridine (P4VP) column combined with SFC can successfully separate the four diastereomers (anomers/epimers) of HexCer (d18:1/16:0) and (d18:1/24:1) at different retention times (RTs) (Fig. 2 A). Thus, these four diastereomers having exactly the same sphingosine and acyl chains could be discriminated for the first time, at least for d18:1/16:0 and d18:1/24:1 species.

**Figure S2.**
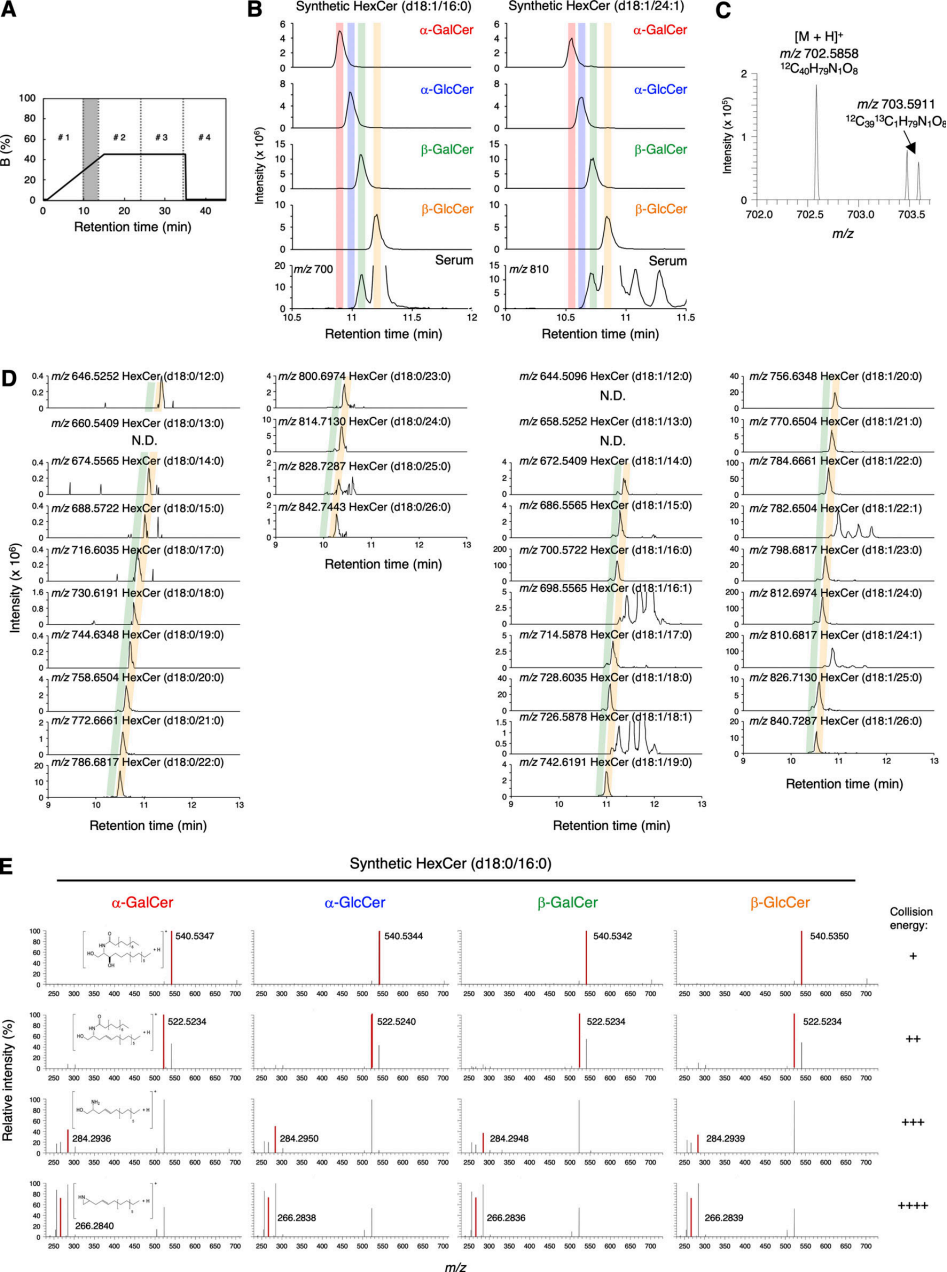
**α-GalCer is detected in serum using SFC/HRMS/MS. (A)** Separation of the C:M = 19:1 fraction from serum by SFC. Samples, including HexCer, with a RT of 9.5–13.5 min were fractionated every 0.5 min. The other parts of the samples were separated into four subfractions: 0–9.5 (#1), 13.5–24 (#2), 24–34.5 (#3), and 34.5–45 min (#4). **(B)** HRMS chromatograms of the synthesized four diastereomers of HexCer (upper) and HexCer in serum (lower) (d18:1/16:0 and d18:1/24:1) obtained using SFC/HRMS. The mass error tolerance of the precursor ions (m/z 700.5722 and m/z 810.6817) was <7 ppm. **(C)** Monoisotopic (m/z 702.5858) and ^13^C_1_ isotopic spectra (m/z 703.5911) of α-GalCer (d18:0/16:0) candidate in serum. **(D)** The HRMS chromatograms were plotted from the theoretical m/z ± 7 ppm of candidate HexCer molecular species in serum obtained using SFC. The colored shadows indicate the peaks coincident with the theoretical RT of β-GlcCer (green) and β-GalCer (yellow), respectively. **(E)** Representative HRMS/MS spectra of four synthesized diastereomers of HexCer (d18:0/16:0) (m/z 702.5878, as [M+H]^+^) obtained using HRMS/MS. From top to bottom, collision energy settings are −10 eV (+), −20 eV (++), −30 eV (+++), and −40 eV (++++). Data are representative of three independent experiments (B–E).

To further purify active subfractions from the C:M = 19:1 fraction, we combined this separation method with an SFC-fraction collector/MRM (SFC-FRC/MRM) system. Judging from the RT of synthetic HexCer, we speculated that fractions around RT 9.5–13.5 min contained HexCer (Fig. S2 A). As subfractions 10–11 min possessed an antigen activity (Fig. 2 B), we analyzed the compounds within this subfraction. RTs of precursor ions of HexCer (d18:1/16:0 and d18:1/24:1) indicated that these HexCer corresponded to β-GlcCer and β-GalCer, but not α anomers (Fig. S2 B). However, a precursor ion of m/z 702.5878, corresponding to HexCer (d18:0/16:0), was detected at RT corresponding to α-GalCer (Fig. 2 C; and Fig. S2, C and D).

**Figure S3.**
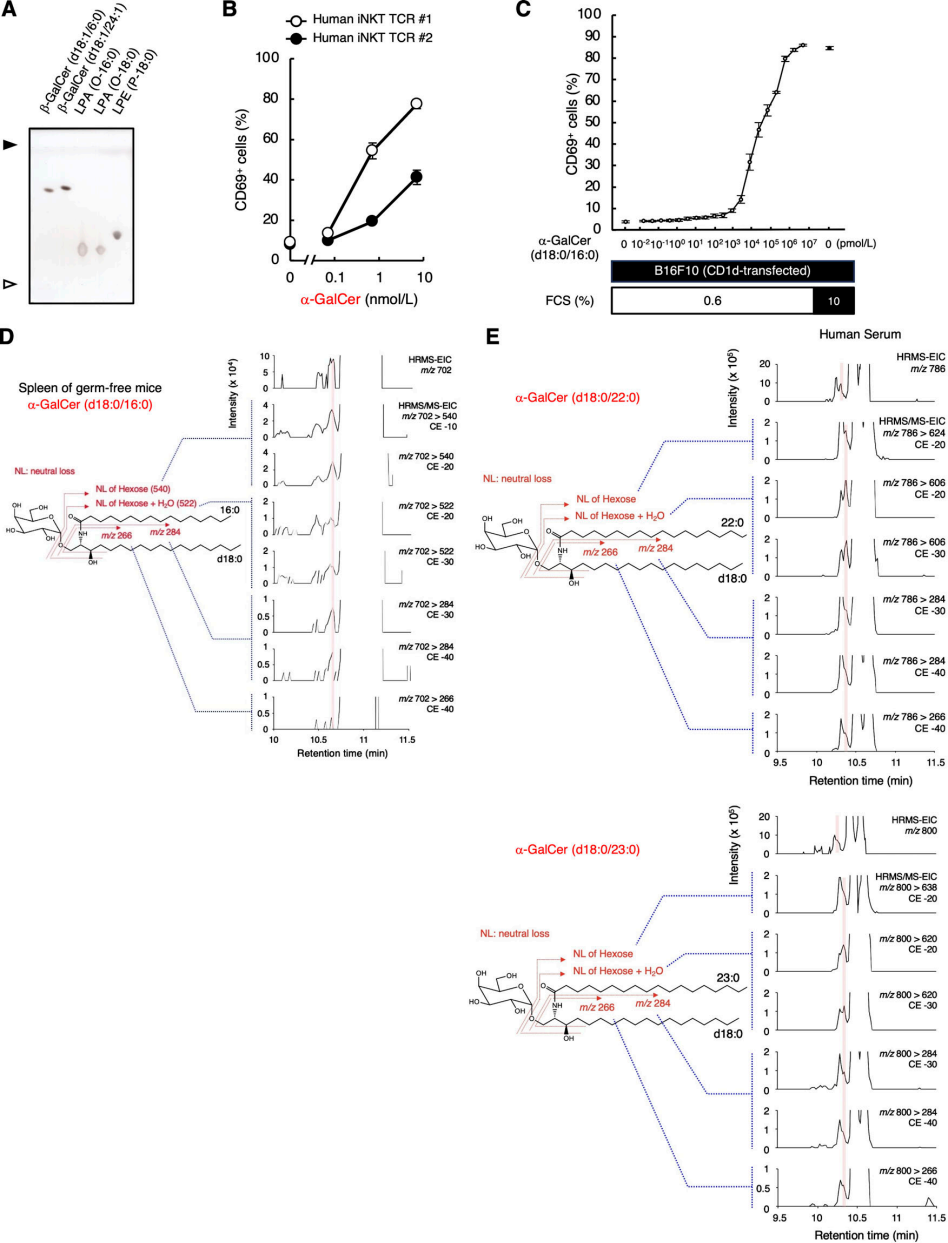
**The presence of α-GalCer in mammalian tissues and fluids. (A)** Synthetic β-GalCer (d18:1/16:0 and d18:1/24:1), LPA (O-16:0 and O-18:0), and LPE (P-18:0) were analyzed by HPTLC using C:M:W (65:25:4; vol/vol/vol) followed by staining with copper acetate reagent. Open and closed arrowheads denote the origin and solvent front, respectively. **(B)** Two clones of human iNKT TCR transfectants (#1 and #2) were stimulated with synthesized α-GalCer (d18:0/16:0) for 16 h and analyzed for CD69 expression. **(C)** CD1d-transduced B16F10 cells were cultured in RPMI 1640 supplemented with 10% FCS or in RPMI 1640 with 0.6% FCS and 9.4% animal component-free cell culture supplement for 7 days. DN32.D3 cells were co-cultured with those B16F10 cells in the presence of indicated concentrations of α-GalCer (d18:0/16:0) for 16 h and analyzed as in B. **(D)** HRMS and HRMS/MS chromatograms of HexCer (d18:0/16:0) in the spleen of germ-free mice. The red shadow indicates the peak coincident with synthesized α-GalCer (d18:0/16:0). **(E)** HRMS and HRMS/MS chromatograms of HexCer detected in human serum by SFC/HRMS/MS. The red shadows indicate the peaks coincident with the theoretical RTs of α-GalCer. The precursor ions as [M+H]^+^ are m/z 786.6817 and 800.6974, respectively. The HRMS chromatograms were plotted from the theoretical m/z ± 7 ppm of candidate HexCer molecular species. Data are presented as mean ± SD (B and C) and are representative of three independent experiments (A–C). CE, collision energy. Source data are available for this figure: SourceData FS3.

**Figure 3.**
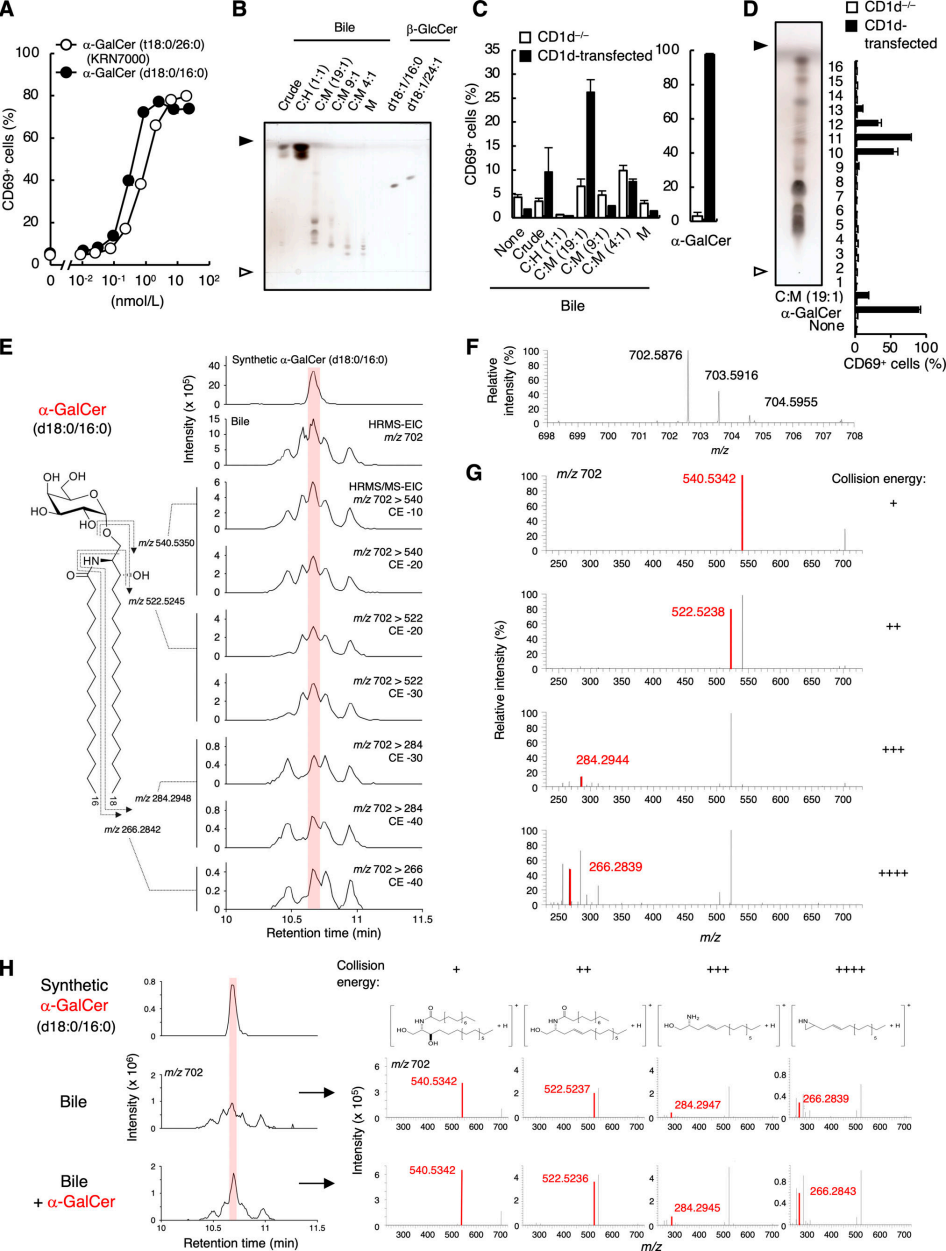
**Detection of α-GalCer in serum and bile. (A)** DN32.D3 cells were stimulated with synthesized α-GalCer (d18:0/16:0) or α-GalCer (t18:0/26:0) (KRN7000) for 16 h and analyzed for CD69 expression. **(B)** Lipid extracts from bovine bile were separated into five fractions by open column chromatography and analyzed by HPTLC using C:M:W (65:25:4; vol/vol/vol) followed by staining with copper acetate reagent. **(C)** CD1d^−/−^ or CD1d-transduced DN32.D3 cells were stimulated for 16 h with each fraction of bovine bile lipids separated in B and analyzed as in A. α-GalCer (t18:0/26:0) was used as a positive control. **(D)** The C:M = 19:1 fraction of bovine bile lipid in B was separated into 16 subfractions by HPTLC (left) and used for stimulation as in C (right). α-GalCer (t18:0/26:0) was used as a positive control. **(E)** HRMS and HRMS/MS chromatograms of HexCer (d18:0/16:0) in bovine bile. **(F and G)** The HRMS spectra (F) and HRMS/MS spectra (G) of candidates for α-GalCer (d18:0/16:0) in bovine bile detected in D. From top to bottom, collision energy settings are −10 eV, −20 eV, −30 eV, and −40 eV (G). **(H)** HRMS chromatograms of HexCer (d18:0/16:0) and the HRMS/MS spectra of candidates for α-GalCer (d18:0/16:0) in bovine bile spiked with synthesized α-GalCer (d18:0/16:0). The area under the curve value ratio of the peak coincident with synthesized α-GalCer (d18:0/16:0) is 4.04 × 10^6^:7.22 × 10^6^. From left to right, collision energy settings are −10 eV (+), −20 eV (++), −30 eV (+++), and −40 eV (++++). The structures of product ions are shown. Open and closed arrowheads denote the origin and solvent front, respectively (B and D). The mass error tolerance of the precursor ion (m/z 702.5878 as [M+H]^+^) was <7 ppm (G and H). The HRMS/MS of precursor-product ion pair: m/z 702.5878 to 540.5350, 522.5245, 284.2948, and 266.2842, respectively (G and H). The red shadow indicates the peak coincident with synthesized α-GalCer (d18:0/16:0) (E and H). Data are presented as mean ± SD (A, C, and D) and are representative of three independent experiments (A–H). CE, collision energy. Source data are available for this figure: SourceData F3.

To verify this assumption, we synthesized α-GlcCer, α-GalCer, β-GlcCer, and β-GalCer (d18:0/16:0) and analyzed them by SFC/high-resolution tandem mass spectrometry (SFC/HRMS/MS), which provides accurate mass measurement of product ions as well as precursor ions for molecular identification. As expected, the minor peak of HexCer (d18:0/16:0) was observed at the same RT with synthetic α-GalCer (d18:0/16:0) (Fig. 2, C and D). Indeed, fragmented ions derived from this RT were identical to that of synthetic α-GalCer (d18:0/16:0) (Fig. 2 E and Fig. S2 E). Furthermore, the intensity of precursor ions and product ions of the estimated α-GalCer (d18:0/16:0) were increased when synthetic α-GalCer (d18:0/16:0) was exogenously spiked into this fraction (Fig. 2 F). The presence of previously reported endogenous iNKT cell antigens, such as vinyl ether-linked lysophosphatidylethanolamine (LPE P, also termed plasmalogen LPE) or ether-linked lysophosphatidic acid (LPA O) (Facciotti et al., 2012), was excluded judging from their relative to front (Rf) values (Fig. S3 A) and MS (data not shown). These results indicate that α-GalCer (d18:0/16:0) was detected in an iNKT-activating fraction from serum. As would be expected from its structure, synthetic α-GalCer (d18:0/16:0) potently activated iNKT cells, similar to the canonical antigen, α-GalCer (t18:0/26:0) (KRN7000) (Fig. 3 A and Fig. S3 B) (von Gerichten et al., 2019; Kawano et al., 1997). Based on the calibration curve for the standard, the amount of α-GalCer (d18:0/16:0) was estimated to be 2.3 fmol/μg in this fraction (Table S2). Without considering any yield loss during the purification processes, at least 18.2 ± 6.5 pM of α-GalCer (d18:0/16:0) is calculated to be present in serum. Indeed, synthetic α-GalCer (d18:0/16:0) could activate iNKT cells at pM orders of concentration within the serum-depleted medium (Fig. S3 C).

### Identification of α-GalCer in mammalian tissues

**Figure 4.**
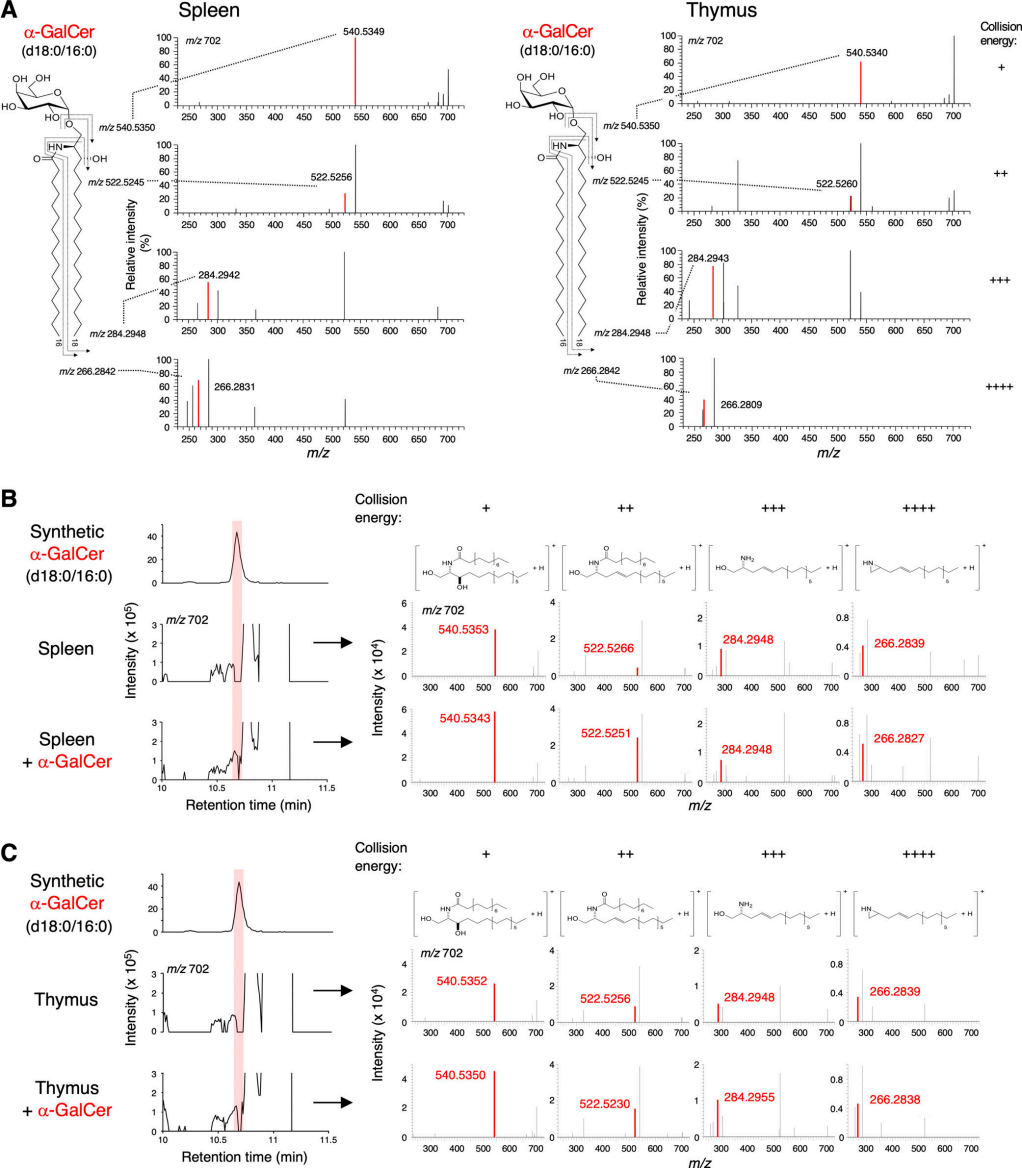
**Detection of α-GalCer (d18:0/16:0) in mammalian tissues. (A)** HRMS/MS spectra of candidate for α-GalCer (d18:0/16:0) (m/z 702.5878, as [M+H]^+^) in mouse spleen (left) and thymus (right). All HRMS/MS mass error tolerances are <7 ppm. From top to bottom, collision energy settings are −10 eV (+), −20 eV (++), −30 eV (+++), and −40 eV (++++). **(B and C)** HRMS chromatograms of HexCer (d18:0/16:0) and the HRMS/MS spectra of candidates for α-GalCer (d18:0/16:0) in spleen (B) and thymus (C) spiked with synthesized α-GalCer (d18:0/16:0). The area under the curve value ratios of each peak coincident with synthesized α-GalCer (d18:0/16:0) are 3.67 × 10^5^:7.35 × 10^5^ (B) and 3.43 × 10^5^:5.94 × 10^5^ (C). The mass error tolerance of the precursor ion (m/z 702.5878 as [M+H]^+^) was <7 ppm (A–C). The HRMS/MS chromatograms of precursor-product ion pair: m/z 702.5878 to 540.5350, 522.5245, 284.2948, and 266.2842, respectively (A–C). The red shadow indicates the peak coincident with synthesized α-GalCer (d18:0/16:0) (B and C). From left to right, collision energy settings are −10 eV (+), −20 eV (++), −30 eV (+++), and −40 eV (++++) (B and C). The structures of product ions are shown (B and C). Data are representative of three independent experiments (A–C).

In addition to serum, we also examined other body fluids and tissues. As iNKT cells are abundantly present in the liver sinusoid around the bile duct (Crosby and Kronenberg, 2018), we analyzed bovine bile. As we detected antigen activity in the C:M = 19:1 fraction (Fig. 3, B and C), we further separated it to determine active subfractions (Fig. 3 D). SFC/HRMS/MS analysis revealed that the peak corresponding to α-GalCer (d18:0/16:0) was detected (Fig. 3, E–G). This assumption was further validated by spike experiments because the intensity of these precursor ion and product ions were clearly and selectively increased by the addition of synthetic α-GalCer (d18:0/16:0) (Fig. 3 H). In addition to bovine bile, we detected α-GalCer (d18:0/16:0) from murine lymphoid organs, such as the thymus and spleen using the same platform and spike validation (Fig. 4, A–C). Of note, α-GalCer (d18:0/16:0) was also detected in the spleen of germ-free mice (Fig. S3 D). These results provide evidence for the presence of α-GalCer in mammals.

**Figure 5.**
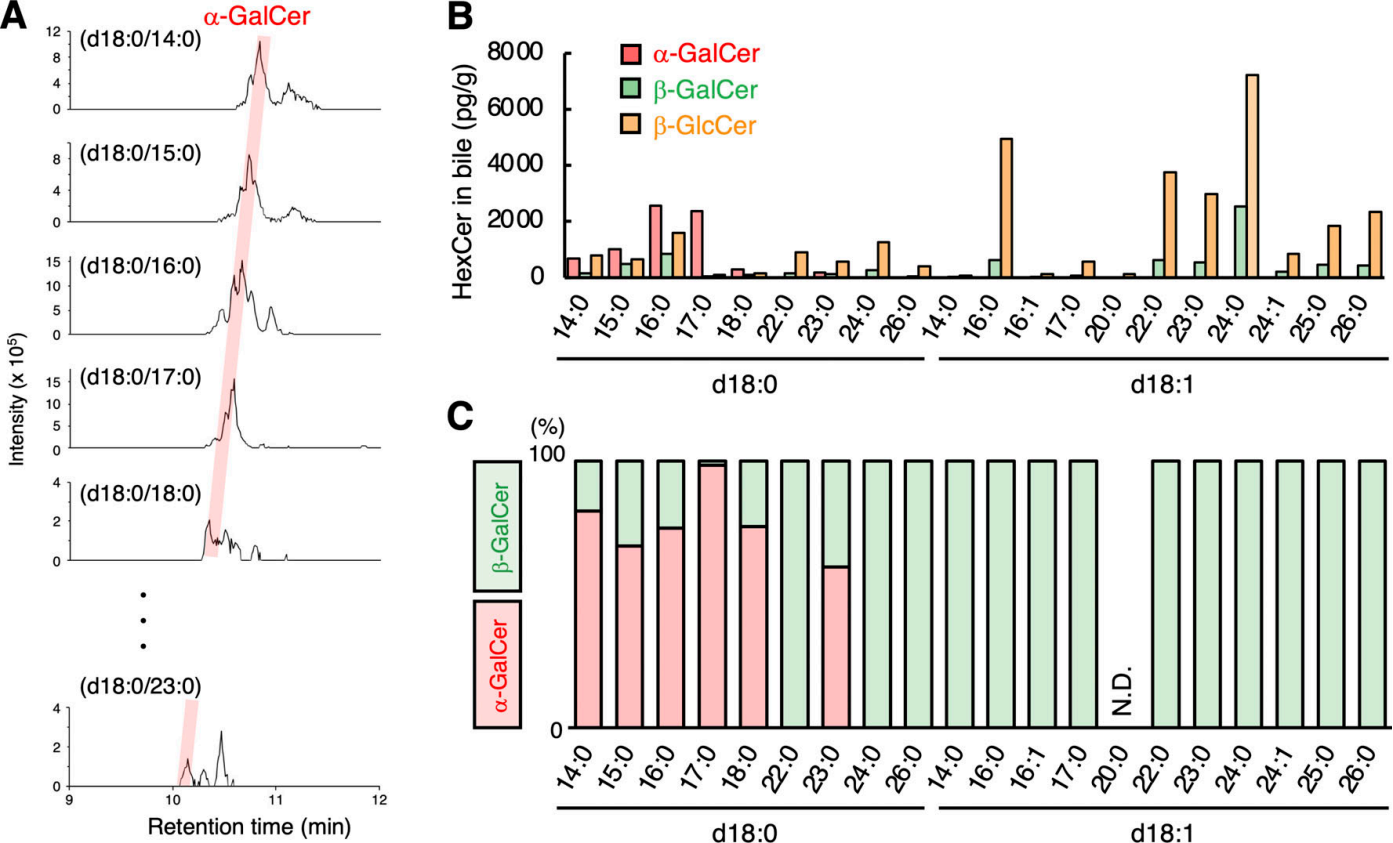
**Detection of α-GalCer (d18:0) species with several lengths of side chains in bile.** **(A)** HRMS chromatograms of HexCer detected in bovine bile by SFC/HRMS/MS. The red shadow indicates the peaks coincident with the theoretical RTs of α-GalCer. The precursor ions as [M+H]^+^ are m/z 674.5565, 688.5722, 702.5878, 716.6035, 730.6191, and 800.6974, respectively. The HRMS chromatograms were plotted from the theoretical *m/z* ± 7 ppm of candidate HexCer molecular species. **(B)** The concentrations of α-GalCer, β-GalCer, and β-GlcCer of indicated species were detected in bovine bile. **(C)** The ratio of α-GalCer to β-GalCer quantities in the indicated GalCer species was detected in bovine bile by SFC/HRMS/MS.

We next asked whether α-GalCer species other than d18:0/16:0 forms are present in bile. By estimating the RT specific to each structure of sphingoid base and acyl side chains using RT trendlines, we attempted to detect various α-GalCer, β-GalCer, and β-GlcCer species (in this trendline analysis α-GlcCer and β-GalCer cannot be clearly separated). Notably, we could detect α-GalCer (d18:0/14:0), (d18:0/15:0), (d18:0/16:0), (d18:0/17:0), (d18:0/18:0), and (d18:0/23:0) (Fig. 5, A–C). α-GalCer species with a sphingoid base (d18:1) were not detected (Fig. 5, B and C). We also detected α-GalCer (d18:0/22:0) and (d18:0/23:0) from human serum (Fig. S3 E). These results suggest that mammals possess dihydrosphingosine-based α-GalCer (d18:0) species with several lengths of saturated side chains.

The separation of four HexCer diastereomers, α-GalCer, α-GlcCer, β-GalCer, and β-GlcCer, has been attempted using hydrophilic interaction LC (Von Gerichten et al., 2017; von Gerichten et al., 2019), although the complete simultaneous separation was not accomplished. In addition, the identification of α-GalCer with MS/MS has been attempted (Brennan et al., 2017; Von Gerichten et al., 2017; von Gerichten et al., 2019). However, as shown by the results of this study, the MS/MS spectrum of α-GalCer in the biological sample was not consistent with that of the synthetic α-GalCer, which is presumably due to the fact that biological samples contain a large amount of matrix, and trace compounds such as α-GalCer are largely influenced by matrix effects in MS/MS fragmentation. Therefore, the identification of α-GalCer by MS/MS spectra only was not accurate, and chromatographic separation was necessary to identify stereoisomers correctly. In this study, we achieved the separation of four HexCer diastereomers using SFC with tandem P4VP columns. Collision-induced dissociation fragmentation patterns were used to discriminate HexCer diastereomers (Brennan et al., 2017; von Gerichten et al., 2019); however, this method could not be generalized in our analysis (Fig. S2 E). Although the cause is currently unknown, the ionic state of [M+Na]^+^ might explain the apparent difference. Taken together, the platform developed in this study is applicable for the determination of various species of HexCer.

Although we could detect antigenic α-GalCer in mammalian tissues, its origin was unknown, as α-specific ceramide galactosyl-transferases have not been reported in mammals thus far (Merrill, 2011; Reza et al., 2021). Hypothetical “unfaithful” reactions of the β-GalCer-synthesizing enzyme, Ugt8a, or putative β to α anomerases that potentially generate trace amounts of α-GalCer (Kim et al., 2014; Kain et al., 2014, 2015) have not been confirmed. Thus, it is currently uncertain whether α-GalCer can be biosynthesized in the host, although we could detect α-GalCer in the spleen of germ-free mice (Fig. S3 D). Importantly, antigenic α-GalCer detected in this study is distinct from recently reported antagonistic/weak branched and hydroxyl α-GalCer derived from symbiotic bacteria (An et al., 2014; Wieland Brown et al., 2013). Alternatively, but not exclusively, α-GalCer may be derived from diet, and small portions of undigested α-GalCer might be transported to the tissues through serum. In the present study, some particular species of α-GalCer (d18:0/14:0, 15:0, 16:0, 17:0, 18:0, and 23:0) were abundantly detected. Given that odd-numbered acyl chains are common in microbes (Heaver et al., 2018), this may provide an implication regarding the origin of α-GalCer in the body.

The molecular mechanisms of thymic selection of iNKT cells are still in debate (Pellicci et al., 2020). In conventional T cells, weak-affinity antigens serve as selecting ligands (Hogquist et al., 1994). Thus, it is unclear whether high-affinity antigen, α-GalCer (Kawano et al., 1997; Sidobre et al., 2002), in the thymus contributes to the thymic development of iNKT cells, although thymic iNKT cells were reported to receive strong TCR signaling (Moran et al., 2011). We cannot exclude the possibility of the involvement of reported iNKT ligands (Gumperz et al., 2000; Zhou et al., 2004; Fox et al., 2009; Facciotti et al., 2012) in the selection of iNKT cells.

In our semiquantitative analysis, the average concentration of α-GalCer in bile is estimated as 10 pmol/g. Furthermore, not all tissues possessed detectable amounts of α-GalCer using our present analytical platform, and thus the precise tissue distribution and abundance of α-GalCer is currently not fully understood. However, as the TCR is a sensitive apparatus that can detect trace amounts of exogenous/endogenous antigens, iNKT cells may recognize host-derived antigens that are locally concentrated on the surface of CD1d-expressing cells. Although the physiological role of host-derived α-GalCer on iNKT cells function is currently unknown, this interaction may constitutively prime iNKT cells for rapid effector function or be involved in tissue residency, particularly in the liver, as the bile duct is close to the sinusoid where iNKT cells are enriched (Crosby and Kronenberg, 2018). Further quantitative analysis and identification of the origin of α-GalCer in the host will advance our insight into the physiological function and biology of unconventional T cells.

Host-derived α-GalCer may play a role in immune cancer surveillance. Given that CD1d-expressing cancer cells were eliminated in an iNKT cell–dependent manner, it is possible that environmental α-GalCer is captured and presented on cancer cells at a functional concentration. As cancer-specific immunotherapy holds great potential, cancer antigens that are specifically expressed in cancer cells have been extensively researched (Haen et al., 2020). The present study may raise the idea that cancer antigens are not necessarily generated in cancer cells. Instead, “environmental” antigen(s), such as lipid metabolites in the body fluids, can be presented. As the efficiency of iNKT activation varied depending on cancer cell lines (Fig. 1 A), it would be of interest to investigate whether a CD1d inducer and the promotion of CD1d-dependent presentation might be an option of antitumor immunotherapy that acts by increasing the availability of environmental antigens.

## Materials and methods

### Mice

C57BL/6J mice were purchased from CLEA Japan. Jα18-deficient mice were provided by M. Taniguchi (RIKEN, Yokohama, Japan). Germ-free mice (C57BL/6NJcl [Gf]) were purchased from CLEA Japan. All mice were maintained in a filtered-air laminar flow enclosure and were provided standard laboratory food and water ad libitum. All animal protocols were approved by the Ethics Committee on Animal Experiments, Research Institute for Microbial Diseases, Osaka University.

### Reagents

α-GalCer (t18:0/26:0) (KRN7000) was purchased from Funakoshi. α-GalCer (d18:1/16:0), β-GalCer (d18:1/16:0), β-GlcCer (d18:1/16:0), α-GalCer (d18:1/24:1), β-GalCer (d18:1/24:1), β-GlcCer (d18:1/24:1), LPE (P-18:0), LPA (O-16:0), and LPA (O-18:0) were from Avanti Polar Lipids. N-stearoyl-D-erythro-sphingosine was from Katayama Chemical Industries Co. Recombinant human glucosylceramidase and recombinant human Galc were from R&D Systems. FCS was from Capricorn Scientific GmbH and Nichirei Biosciences Inc. XerumFree was from TNC BIO BV. Bovine bile was provided by M. Yasuda (Miyazaki University, Miyazaki, Japan). Anti-mouse CD1d antibody (1B1) was from BD Biosciences. Anti-mouse CD69 antibody (H1.2F3) and rat IgG2b, κ isotype control (RTK4530) were purchased from BioLegend. Enzyme-linked immunosorbent assay (ELISA) kit for mouse interleukin-2 (IL-2) was from BD Biosciences.

### Cells

DN32.D3 (Lantz and Bendelac, 1994) cells were provided by Y. Kinjo (Jikei University School of Medicine, Tokyo, Japan) and 2B4 cells (Chien et al., 1984) were provided by T. Saito (RIKEN, Yokohama, Japan). B16F10 cells were from RIKEN BioResource Research Center and MC38 cells were from kerafast. A549, MDA-MB-231, PANC-1, EL4, and LLC1 cells were from the American Type Culture Collection. Cells were cultured in RPMI 1640 or DMEM supplemented with 10% FCS, penicillin (Sigma-Aldrich), streptomycin (MP Biomedicals), and 2-mercaptoethanol (Nacalai Tesque). DN32.D3, B16F10, MC38, 2B4, HEK293, HeLa, A549, MDA-MB-231, PANC-1, EL4, and LLC1 cells were transfected with mouse CD1d using retrovirus-mediated gene transfer (pMX-IRES-GFP, pMXs-puro or pMX-Neo). Cells deficient in CD1d, TCR (Vα14), Ugcg, and Ugt8a were generated by the CRISPR/Cas9 system using pX330 or lentiCRISPR v2 plasmids (Addgene). Single clones were isolated by limiting dilution and were genotyped by sequencing the single guide RNA target region of the corresponding chromosomal gene. Mouse T cell hybridomas with an NFAT-GFP reporter gene were transfected with human CD1d and synthesized human iNKT TCRα and β chain cDNA sequences to reconstitute TCRαβ pairs (Matsumoto et al., 2021; Lu et al., 2021) using retrovirus-mediated gene transfer (pMX-IRES-human CD8, pMX-IRES-rat CD2). The usages of V and J gene segments and CDR3 amino acid sequences of the α and β chains of human iNKT cells are as follows: #1 TRAV10/TRAJ18/CDR3α (CVVSDRGSTLGRLYF) and TRBV25-1/TRBJ2-2/CDR3β (CASSEPPGRELFF), #2 TRAV10/TRAJ18/CDR3α (CVVSDRGSTLGRLYF) and TRBV25-1/TRBJ2-1/CDR3β (CASSSGTPDNEQFF).

### Lipid extraction and purification

Lipids were extracted using a modified Folch method (Bligh and Dyer, 1959). Briefly, the samples were treated with C:M:water (W) (6:3:1, vol/vol/vol) for 1 day. The mixture was separated by centrifugation, and the lower organic phase was collected as the lipid extract. After filtration using Millex-LG 0.2 μm (Merck), the lipid extracts were visualized by HPTLC (Merck) using C:M:W (65:25:4, vol/vol/vol) and copper (II) acetate–phosphoric acid staining (180°C, 5 min).

### Lipid fractionation by open-column chromatography

The extracted lipids were resuspended in hexane:chloroform (1:1, vol/vol) and applied to InertSep SI (GL Sciences) equilibrated with hexane:chloroform (1:1, vol/vol). These were eluted using a stepwise gradient of eluate composition, starting with hexane:chloroform (1:1, vol/vol) and then chloroform, C:M (49:1, vol/vol), C:M (19:1, vol/vol), C:M (9:1, vol/vol), C:M (4:1, vol/vol), and M according to the manufacturer’s instructions. The fractions were evaporated and resuspended in C:M (2:1, vol/vol).

### Treatment of lipids

For hydrolysis treatment using sodium hydroxide, the lipids were hydrolyzed for 3 h at 37°C in C:M (2:1, vol/vol) containing 1 M NaOH. 0.5 vol of W was added to the treated sample. Then, the sample was separated by centrifugation and the lower organic phase was collected.

For Gba treatment, lipids were resuspended in 50 mM sodium dihydrogen citrate buffer (pH 5.5) containing 0.25% sodium taurocholate and 5 mM dithiothreitol. For Galc treatment, lipids were resuspended in 50 mM sodium dihydrogen citrate/200 mM sodium hydrogen phosphate/150 mM sodium chloride buffer (pH 4.3) containing 0.25% sodium taurochorate and 5 mM dithiothreitol. Gba was added at a concentration of 293 μg/ml/mg lipid and Galc was added at a concentration of 136 μg/ml/mg lipid respectively, and digestion was performed for 16 h at 37°C in a shaker. After digestion, 4 vol of W and 30 vol of C:M (2:1, vol/vol) were added. The lower organic phase was collected after centrifugation.

### In vitro stimulation assay

To stimulate DN32.D3 cells, each lipid was diluted in isopropanol, and 80 μl of dilutions were added to each well of a 96-well plate, followed by evaporation of the solvent. The plate was vigorously shaken using a microplate mixer for 10 min after adding 100 μl of 10% RPMI 1640, and 5 × 10^5^ cells of CD1d^−/−^ or CD1d-expressing DN32.D3 cells were added and cultured in 200 μl for 18 h at 37°C. For coculture assay in low FCS concentrations, cells were cultured in 200 μl of RPMI 1640 with 0.6% FCS and 9.4% XerumFree XF212 (TNC Bio) for 18 h at 37°C. The expression of CD69 on DN32.D3 cells was analyzed using a FACSCalibur flow cytometer (BD Biosciences) or Attune NxT flow cytometer (Thermo Fisher Scientific). IL-2 levels in the culture supernatants were analyzed by ELISA (BD Biosciences).

### Tumor challenge

B16F10 cells (5 × 10^5^) suspended in phosphate-buffered saline were inoculated subcutaneously into the right flanks of mice. Tumor diameter was monitored and tumor volume was calculated using the following formula: width × length × height × 0.52 (Cleave et al., 1991).

### LC-FRC

B16 cells were prepared for lipid extraction using the Bligh and Dyer method (Bligh and Dyer, 1959). Briefly, the lipids were extracted from cells with 1,000 μl of methanol and 400 μl of chloroform. The samples were vigorously mixed for 1 min and sonicated for 5 min. The extracts were then centrifuged at 16,000 × *g* for 5 min at 4°C and the resultant supernatant was collected. The collected supernatant (700 μl) was mixed with 300 μl of chloroform and 400 μl of water, and then centrifuged at 16,000 × *g* for 5 min at 4°C. The organic layer (350 μl) was dried under nitrogen and stored at −80°C until analysis.

The separation and fractionation of the extracted lipids of B16 cells were performed using a Nexera X2 system (Shimadzu Corp.) coupled to TriVersa NanoMate (Advion).

The LC analysis conditions were as follows: column, InertSustain C18 (2.1 inner diameter [i.d.] × 150 mm, 3 μm particle size; GL Sciences); column temperature, 50°C; mobile phase A, W:acetonitrile (1:2, vol/vol) with 5 mM ammonium acetate; mobile phase B, M:isopropanol (1:19, vol/vol) with 5 mM ammonium acetate; and flow rate, 0.2 ml min^−1^. The gradient conditions were as follows: 0–100% B, 74 min; 100% B, 10 min; 100–0% B, 0.1 min; and 0% B, 6 min. Sample fractions were performed in 96-well plates at 1-min intervals from 0 to 84 min of the total 90-min LC analysis.

### SFC/MRM analysis

Lipid extraction of WT and mutated B16 cells were identical to those of the LC-FRC. The HexCer analysis was performed using a Nexera UC system (Shimadzu Corp.) coupled to an LCMS-8060 system (Shimadzu Corp.) (SFC/MRM).

The SFC analysis conditions were as follows: column, two metal-free polyether ether ketone (PEEK)–coated P4VP columns (2.1 × 150 mm, 3.0 μm; Daicel Corp.); column temperature, 40°C; mobile phase A, supercritical carbon dioxide; mobile phase B, M:W (95:5, vol/vol) with 0.1% (wt/vol) ammonium acetate; flow rate of mobile phase, 0.8 ml min^−1^; and flow rate of make-up pump, 0.1 ml min^−1^; back-pressure regulator, 10 MPa. The gradient conditions were as follows: 1% B, 0–1 min; 1–45% B, 1–15 min; 45% B, 15–35 min; and 1% B, 35–45 min. Two metal-free PEEK-coated P4VP columns were connected in series.

The MRM operating conditions were as follows: polarity, positive ionization; electrospray voltage, 4.0 kV; desolvation line temperature, 250°C; heat block temperature, 400°C; nebulizing gas flow rate, 3.0 L min^−1^; drying gas flow rate, 10.0 L min^−1^; collision-induced dissociation gas pressure, 0.23 MPa; and detector voltage, 2.16 kV; dwell time, 2 ms; pause time, 2 ms. Data processing was performed using LabSolution software v5.99 SP2 (Shimadzu Corp.).

### SFC-FRC/MRM analysis

The separation, fractionation, and analysis of the C:M = 19:1 fractions of FCS were performed using a Nexera UC system (Shimadzu Corp.) coupled to an LCMS-8060 system (Shimadzu Corp.) equipped with FRC-40 (Shimadzu Corp.) (SFC-FRC/MRM).

The SFC analytical conditions were identical to those of the SFC/MRM analysis method with modifications as follows: column, three metal-free PEEK-coated P4VP columns (2.1 × 150 mm, 3.0 μm; Daicel Corp.); flow rate of mobile phase, 0.6 ml min^−1^. Three metal-free PEEK-coated P4VP columns were connected in series.

The FRC operating conditions were as follows: make-up, isopropanol; flow rate of make-up pump for collecting the fraction samples, 3.0 ml min^−1^.

The MRM operating conditions were identical to those of the SFC/MRM analysis method.

### SFC/HRMS/MS analysis for structural analysis

The identification of HexCer species was performed using a Nexera UC system (Shimadzu Corp.) coupled to an Orbitrap Exploris 120 Mass Spectrometer (Thermo Fisher Scientific) with a heated electrospray ionization (ESI) source (Thermo Fisher Scientific) (SFC/HRMS/MS).

The SFC analytical conditions were identical to those of the SFC-FRC/MRM analysis method.

The HRMS/MS operating conditions were as follows: polarity, positive ion mode; sheath gas flow rate, 10 arb for positive ionization, Aux gas flow rate, 0.5 arb; spray voltage, 3.5 kV for positive ionization; ion transfer tube temperature, 320°C; vaporizer temperature, 100°C; radio-frequency (RF) lens, 70%; Orbitrap resolution, 60,000; automatic gain control (AGC) target, 10,000,000; maximum injection (MI) time, 200 ms; and scan range, 200–1,200 (m/z). The parallel reaction monitoring conditions for each target compound were as follows: positive ionization; Orbitrap resolution, 15,000; AGC target, 3,000,000; MI time, 100 ms; Q1 resolution, 0.4 (m/z); normalized collision energy, −10, −20, −30, −40 eV (fixed); and scan range mode, auto. Data processing was performed using Xcalibur software (Thermo Fisher Scientific).

### Spike approach

A standard spike test was carried out for each mammalian sample by adding the following amount of synthetic α-GalCer (d18:0/16:0) standard; 0.61 pmol on column for FSC sub-fraction; 0.21 pmol on column for mouse thymus sub-fraction; 0.20 pmol on column for mouse spleen sub-fraction; and 0.27 pmol on column for bovine bile.

### Chemical synthesis

The HexCer including α-/β-GalCer (d18:0/16:0) and α-GlcCer (d18:0/16:0, d18:1/24:1 [15Z], d18:1/16:0) were chemically synthesized according to the following synthetic sequences, respectively. α-/β-GalCer (d18:0/16:0) were prepared starting from the same galactose derivative, phenyl 4,6-*O*-benzylidene-2,3-di-*O*-*tert*-butylbenzyl-1-thio-β-D-galactopyranoside. First, the thioglycoside was glycosidated with (2*S*,3*R*,4*E*)-2-azido-3-*O*-benzoyl-4-octadecene-1,3-diol in the presence of dimethyl(methylthio)sulfonium trifluoromethanesulfonate (DMTST) and 2,4,6-tri-*tert*-butylpyrimidine (TTBP) in CH_2_Cl_2_ at 0°C, affording galactosyl azido-sphingosine (GalSph) derivatives in total 76% yield (α-glycoside: 56%, β-glycoside: 20%). The respective α- and β-GalSphs were separately treated with NaOMe in a mixed solvent of MeOH and tetrahydrofuran (THF) to remove the benzoyl group on the sphingosine moiety. Both debenzoylated products were then subjected to hydrogenation for reduction of the azido group and of the double bond, and concomitant removal of the benzylidene and *tert*-butylbenzyl groups was followed by the reaction with palmitoyl chloride and Et_3_N in MeOH, yielding the target α-GalCer (d18:0/16:0) and β-GalCer (d18:0/16:0), respectively. Next, the synthesis of α-/β-GlcCer (d18:0/16:0) was started from the coupling of phenyl 4,6-*O*-benzylidene-2,3-di-*O*-*tert*-butylbenzyl-1-thio-β-D-glucopyranoside and (2*S*,3*R*,4*E*)-2-azido-3-*O*-benzoyl-4-octadecene-1,3-diol in the presence of DMTST and TTBP in CH_2_Cl_2_ at 0°C, affording the crude glucosyl azido-sphingosine (GlcSph) derivatives as a mixture of stereoisomers in 95% yield. The removal of the two *tert*-butylbenzyl groups was conducted with 2,3-dichloro-5,6-dicyano-*p*-benzoquinone (DDQ) and phosphate buffer in CH_2_Cl_2_ to give the deprotected compound, and the two anomers were successfully separated at this stage. The benzoyl group on the sphingosine moiety was then removed by the action of NaOMe in MeOH. Each GlcSph anomer was separately subjected to the reduction of the azido group with zinc and saturated aqueous (Satd. aq.) NH_4_Cl in MeOH followed by the coupling with palmitic acid in the presence of 4-(4,6-dimethoxy-1,3,5-triazin-2-yl)-4-methylmorpholinium chloride (DMTMM) in MeOH, affording α- and β-glucosyl ceramides, respectively. Finally, hydrogenation with palladium (Pd)/C in a mixed solvent of EtOAc and MeOH under a hydrogen atmosphere furnished the target α-GlcCer (d18:0/16:0) and β-GlcCer (d18:0/16:0). Next, other α-GlcCer, d18:1/24:1 (15Z) and d18:1/16:0 were prepared by an indirect synthetic approach starting from a galactose derivative. The known 4,6-*O*-di-*tert*-butylsilylene (DTBS)-protected galactosyl donor, 2,3-di-*O*-benzoyl-4,6-*O*-DTBS-α-D-galactopyranosyl trichloroacetimidate was glycosidated with (2*S*,3*R*,4*E*)-3-*O*-*p*-methoxybenzyl-2-tetrachlorophthalimido-4-octadecene-1,3-diol in the presence of trimethylsilyl trifluoromethanesulfonate (TMSOTf) in CH_2_Cl_2_ at 0°C, giving α-GalSph predominantly that can be easily separated by flash chromatography (Imamura et al., 2003, 2016). The DTBS group was then removed by the action of tributylammonium hydrogenfluoride. Subsequently, regioselective benzoylation at the C6 position of Gal residue was performed using Bz_2_O and 4-dimethylaminopyridine (DMAP) in pyridine at −40°C to give 4-OH GalSph. Epimerization of galactopyranoside to the corresponding glucopyranoside structure was conducted by triflation with Tf_2_O and pyridine in CH_2_Cl_2_ at 0°C followed by the treatment with CsOAc in MeCN, affording the desired α-GlcSph in 80% yield over two steps. Subsequent removal of the acyl and tetrachlorophthaloyl protecting groups gave a fully unprotected α-GlcSph derivative. Finally, the coupling of the α-GlcSph having the liberated C2-amine on the sphingosine moiety and palmitic acid/nervonic acid was performed in the presence of DMTMM in MeOH, yielding the target α-GlcCer (d18:1/24:1 (15Z) and d18:1/16:0).

### Statistical analysis

An unpaired two-tailed Student’s *t* test was used for all statistical analyses. Asterisks denote the level of statistical significance (*P < 0.05).

### Online supplemental material

Fig. S1 shows the presence of antigens for iNKT cells in serum. Fig. S2 shows the detection of α-GalCer in serum using SFC/HRMS/MS. Fig. S3 shows the presence of α-GalCer in mammalian tissues and fluids. Table S1 shows the list of columns used for separation of HexCer diastereomers. Table S2 shows the detection sensitivities for α-GalCer by SFC/HRMS/MS and SFC/MRM.

## Supplementary Material

Table S1 shows the list of columns for screening to evaluate separation of HexCer diastereomers.

Table S2 shows the detection sensitivities for α-GalCer (d18:0/16:0) using SFC/HRMS/MS and SFC/MRM.

SourceData F1 is the source file for Fig. 1.

SourceData F3 is the source file for Fig. 3.

SourceData FS1 is the source file for Fig. S1.

SourceData FS3 is the source file for Fig. S3.

## Data Availability

All data are available in the article itself and its supplementary materials and are also available upon request from the corresponding authors.
