# Supplementary material for: Identification of α-galactosylceramide as an endogenous mammalian antigen for iNKT cells
Source: J Exp Med. 2024 Dec 20;222(2):e20240728. doi: 10.1084/jem.20240728 (PMC11660903; doi:10.1084/jem.20240728)
Supplement: Table S1 — shows the list of columns for screening to evaluate separation of HexCer diastereomers. [file jem_20240728_tables1.docx]

**Table S1. The list of columns for screening to evaluate separation of HexCer diastereomers. Related to Figure 2.**

| Company | Name | Column coating | Particle size (μm) | Inner diameter (mm) | Length (mm) |
| --- | --- | --- | --- | --- | --- |
| Waters Corp. | Torus 1-AA | SUS | 1.7 | 3 | 100 |
|  | Torus 2-PIC | SUS | 1.7 | 3 | 100 |
|  | Viridis BEH 2-EP | SUS | 1.7 | 3 | 100 |
|  | Viridis BEH | SUS | 1.7 | 3 | 100 |
|  | Torus DEA | SUS | 1.7 | 3 | 100 |
|  | Torus Diol | SUS | 1.7 | 3 | 100 |
| Daicel Corp. | DCpak P4VP | SUS | 3 | 2.1 | 150 |
|  | DCpak P4VP | PEEK | 3 | 2.1 | 150 |
|  | DCpak PTZ | SUS | 3 | 2.1 | 150 |
|  | DCpak PTZ | PEEK | 3 | 2.1 | 150 |
|  | CHIRALPAK IA-3 | SUS | 3 | 3 | 150 |
|  | CHIRALPAK IB-3 | SUS | 3 | 3 | 150 |
|  | CHIRALPAK IC-3 | SUS | 3 | 3 | 150 |
|  | CHIRALPAK ID-3 | SUS | 3 | 3 | 150 |
|  | CHIRALPAK IE-3 | SUS | 3 | 3 | 150 |
|  | CHIRALPAK IF-3 | SUS | 3 | 3 | 150 |
|  | CHIRALPAK IH-3 | SUS | 3 | 3 | 150 |
|  | CHIRALPAK AD-3 | SUS | 3 | 3 | 150 |
|  | CHIRALPAK AY-3 | SUS | 3 | 3 | 150 |
|  | CHIRALPAK OD-3 | SUS | 3 | 3 | 150 |

SUS, steel use stainless; PEEK, polyether ether ketone.
