# Supplementary material for: Identification of α-galactosylceramide as an endogenous mammalian antigen for iNKT cells
Source: J Exp Med. 2024 Dec 20;222(2):e20240728. doi: 10.1084/jem.20240728 (PMC11660903; doi:10.1084/jem.20240728)
Supplement: Table S2 — shows the detection sensitivities for α-GalCer (d18:0/16:0) using SFC/HRMS/MS and SFC/MRM. [file jem_20240728_tables2.docx]

**Table S2. The detection sensitivities for α-GalCer (d18:0/16:0) using SFC/HRMS/MS and SFC/MRM.**

**Related to Figure 2 and 3.**

| Species | Method | Ion mode | Targeted ion (*m/z*) | Retention time (min) | Lod (fmol)^a^ | Liner range (μM) | *R*^2^ |
| --- | --- | --- | --- | --- | --- | --- | --- |
| α-GalCer  (d18:0/16:0) | SFC/HRMS/MS | ESI+ | Precursor ion: 702.5878 | 10.67 ± 0.01 | 13 | 0.010−1.0 | 0.9981 |
|  | SFC/MRM |  | MRM transition: 702.59 > 266.28 | 11.25 ± 0.01 | 50 | 0.050−1.0 | 0.997 |

^a^LOD (limit of detection) was estimated based on *S/N* = 3.
