# Supplementary figures and images for: Identification of α-galactosylceramide as an endogenous mammalian antigen for iNKT cells
Source: J Exp Med. 2024 Dec 20;222(2):e20240728. doi: 10.1084/jem.20240728 (PMC11660903; doi:10.1084/jem.20240728)

# Source data for Fig. 1

Fig. 1F

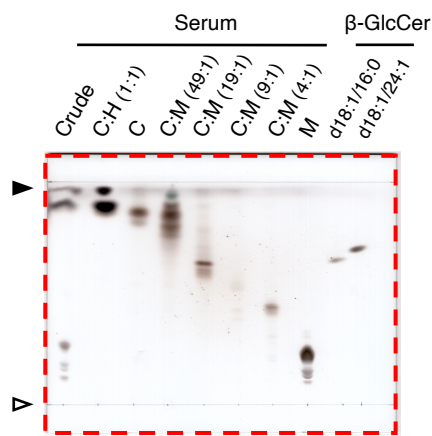

Supplement: SourceData F1 — is the source file for Fig. 1. [file jem_20240728_sourcedataf1.pdf]

Source data for Fig. 3

Fig. 3B

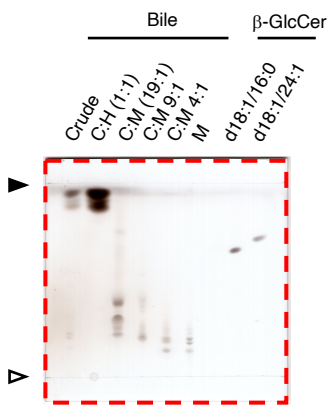

Fig. 3D

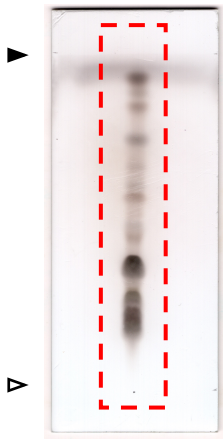

Supplement: SourceData F3 — is the source file for Fig. 3. [file jem_20240728_sourcedataf3.pdf]

# Source data for Supplemental Fig. 1

Fig. S1E

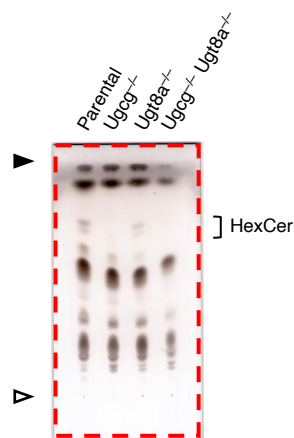

Fig. S1G

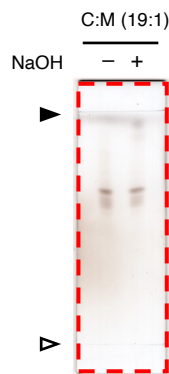

Fig. S1H

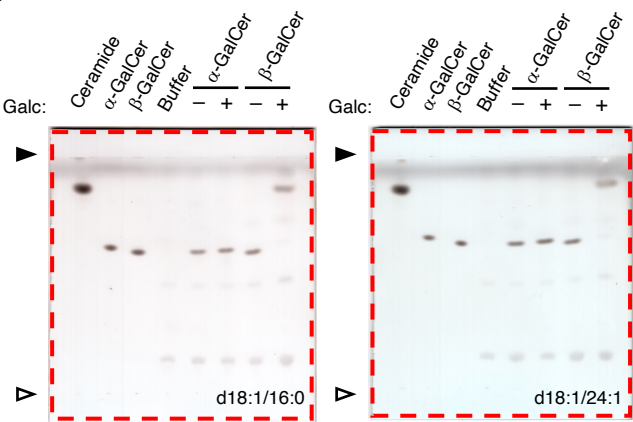

Fig. S1I

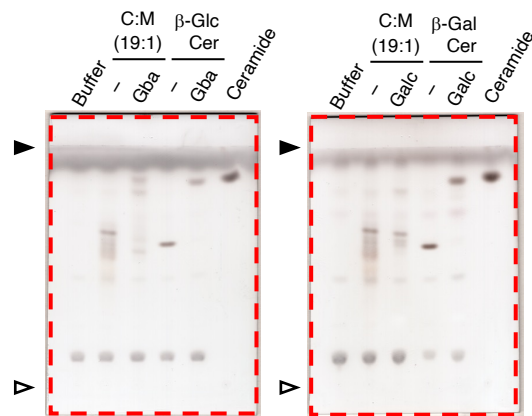

Supplement: SourceData FS1 — is the source file for Fig. S1. [file jem_20240728_sourcedatafs1.pdf]

Source data for Supplemental Fig. 3

Fig. S3A

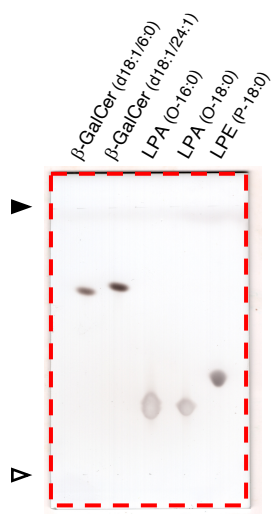

Supplement: SourceData FS3 — is the source file for Fig. S3. [file jem_20240728_sourcedatafs3.pdf]
